# PROTOCOL: Mindfulness‐based interventions for improving tic‐related symptoms in children and adults with chronic tic disorder and Tourette's disorder: A systematic review

**DOI:** 10.1002/cl2.1427

**Published:** 2024-07-21

**Authors:** Ilana Singer, Méliza Gagnon, Julie B. Leclerc

**Affiliations:** ^1^ Department of Psychology Université du Québec à Montréal Montreal Quebec Canada; ^2^ Centre de recherche du CIUSSS du Nord‐de‐l’île‐de‐Montréal Montreal Quebec Canada; ^3^ Centre de recherche de l'Institut universitaire en santé mentale de Montréal Montreal Quebec Canada

**Keywords:** adults, children, intervention, mindfulness, therapy, tics, Tourette

## Abstract

This is a protocol for a Cochrane Review (prototype). The objectives are as follows: This systematic review aims to summarize the available scientific literature on the effect of mindfulness‐based approaches in children and adults with Chronic Tic Disorder (CTD) or Tourette Disorder (TD). More specifically, the research question is: What is the effectiveness of mindfulness‐based interventions in reducing tic severity and improving overall functioning, quality of life, and co‐occurring symptoms in children and adults with CTD or TD?

## 1 BACKGROUND

### 1.1 The problem, condition or issue

Chronic Tic Disorder (CTD) and Gilles de la Tourette's Disorder (TD) are neurodevelopmental conditions (American Psychiatric Association, 2022). CTD is characterized by the presence of motor or auditory tics and TD is characterized by the presence of several motor tics and at least one auditory tic. In both cases, tics persist for at least 1 year and appear before the age of 18 (American Psychiatric Association, 2022). Tics are repetitive, sudden, involuntary, and non‐rhythmic movements or sounds (American Psychiatric Association, 2022). Tics can be classified as simple or complex. Simple tics involve a single muscle group, such as blinking (motor tic) or throat clearing (sound tic). Complex tics involve several muscle groups, for example echopraxia (motor tic), which involves involuntarily imitating the movements of others, or palilalia (sound tic), which involves involuntarily repeating words or phrases (American Psychiatric Association, 2022).

The prevalence of CTD and TD is respectively established at around 1.87% and around 0.75% (Knight et al., 2012; Scahill et al., 2013; Yang et al., 2016). The onset of tics is most frequent between the ages of 4 and 6, with a peak in severity around age 11 (American Psychiatric Association, 2022). Tics are 2 to 4 times more frequent in boys than in girls (American Psychiatric Association, 2022). Although the frequency of tics often stabilizes or decreases after adolescence, between 30% and 50% of individuals still have persistent tics in adulthood (American Psychiatric Association, 2022; Gill & Kompoliti, 2020). In addition, around 80% of people with both CTD and TD have co‐occurring difficulties, such as attention deficit disorder with or without hyperactivity (ADD/ADHD), obsessive‐compulsive disorder (OCD), anxiety and depression (Eapen et al., 2016; Gill & Kompoliti, 2020; Solis‐Garcia et al., 2021). As a result, children and adults often face issues that lead to feelings of isolation, diminished self‐esteem, and difficulties in academic or work environments (Cox et al., 2019; Eapen et al., 2016). Such issues have a tangible effect and often lead to a significant decline in their overall quality of life (Nolin & Leclerc, 2021).

Despite behavioral interventions such as Habit Reversal Training (HRT), Comprehensive Behavioral Intervention for Tics (CBIT), and Exposure Response Prevention (ERP) being recommended for tic management (Andrén et al., 2022; Pringsheim et al., 2019), a notable fraction of individuals do not achieve sustained improvement over time (Scahill et al., 2013; Wilhelm et al., 2012). This therapeutic gap necessitates exploring alternative interventions. Mindfulness‐based therapies, effective in mitigating anxiety and depression — conditions often co‐morbid with CTD and TD — offer promising avenues. Mindfulness focuses on cultivating self‐awareness and emotional regulation (Haller et al., 2021; Kabat‐Zinn, 1982), and can potentially mitigate factors that exacerbate tics (Conelea & Woods, 2008). To date, no comprehensive systematic review has assessed the potential of mindfulness‐based interventions (MBIs) in the CTD and TD population.

### 1.2 The intervention

Mindfulness has its origins in ancient Eastern traditions (Kabat‐Zinn, 1982). This approach is increasingly integrated into cognitive‐behavioral therapies (CBT). Mindfulness enables individuals to carefully observe their thoughts, emotions, and bodily sensations (Kabat‐Zinn, 1982). These practices not only improve self‐awareness, but also promote emotional regulation and adaptability (Kabat‐Zinn, 1982). *Mindfulness‐Based Stress Reduction* (MBSR; Kabat‐Zinn, 1990, 2003) was one of the first emerging therapies. MBSR combines body awareness with yoga practices to reduce stress and improve overall well‐being (Kabat‐Zinn, 1990, 2003). *Dialectical Behavior Therapy* (DBT) and *Acceptance and Commitment Therapy* (ACT) were then introduced (Hayes et al., 1999; Heard & Linehan, 1994). DBT uses mindfulness to develop emotional regulation and interpersonal skills (Heard & Linehan, 1994). ACT uses mindfulness to promote acceptance and behavior change (Hayes et al., 1999). In addition, in the early 2000s, *Mindfulness‐Based Cognitive Therapy* (MBCT; Morgan, 2003) was introduced. MBCT combines mindfulness practices with CBT (Morgan, 2003). This development underlines the emergence of a “third wave” of CBT, which represents an advance on conventional therapeutic methods (Dimidjian et al., 2016).

MBSR, developed by Kabat‐Zinn (1982), is a program focused on reducing stress and enhancing well‐being through mindfulness meditation, body awareness, and yoga. Spanning typically 8 weeks, MBSR is designed to heighten individuals' awareness of their thoughts, feelings, and bodily sensations, thereby aiding them in managing stress and associated symptoms more effectively. DBT, created by Marsha Linehan (1994), integrates mindfulness as a fundamental element to foster emotional regulation and enhance interpersonal skills. Originally developed for individuals with borderline personality disorder, DBT has since been adapted for a range of other conditions. Its core principle is to strike a balance between acceptance and change, equipping individuals with the skills needed to manage intense emotions and foster healthier relationships. ACT, developed by Hayes and colleagues (1999), employs mindfulness to cultivate psychological flexibility. ACT focuses on encouraging individuals to accept their unpleasant feelings and commit to acting in accordance with their personal values. This approach aims to help individuals engage in value‐aligned behaviors, thereby improving their overall quality of life. Lastly, MBCT is an innovative intervention that merges traditional CBT techniques with mindfulness practices. Developed by Zindel Segal, Mark Williams, and John Teasdale (2003), MBCT particularly assists individuals in becoming more conscious of and altering their relationship with negative thought patterns.

A typical MBI session might include guided mindfulness exercises focusing on non‐judgmental awareness of present sensations and thoughts (Allen et al., 2021). Such practices are particularly beneficial for individuals with tics as they are tailored to address the triggers and experiences unique to each condition. Variations in the delivery of MBIs allow these interventions to cater to a wide range of needs and preferences. This includes individual therapy sessions, group workshops, and digital platforms, offering diverse avenues for individuals to engage in mindfulness practices (Allen et al., 2021). The delivery of MBIs are typically overseen by psychologists or therapists with specialized training in mindfulness practices. Standard MBI programs generally span an 8‐week period, with each session lasting approximately 60–90 min (Allen et al., 2021). This structure provides a consistent framework while allowing for the flexibility needed to tailor the program to the individual's unique symptomatology and life circumstances. Supplementary practices and exercises are often recommended for daily practice outside formal sessions, reinforcing the skills and strategies learned during the sessions and promoting continued personal growth and symptom management.

### 1.3 How the intervention might work

MBIs are increasingly validated by research. A review of the literature highlighted the benefits of MBSR, ACT, DBT and MBCT in the treatment of anxiety and depression in adults (Haller et al., 2021). Specifically, MBSR showed superior short‐term effects compared with CBT on anxiety (*n* = 218, standard mean difference [SMD] = −1.42) and depressive symptoms (*n* = 218, SMD = −1.13; Haller et al., 2021). ACT showed significant short‐term benefits over second‐wave CBT in treating anxiety (*n* = 204, SMD = −0.98,) and depressive symptoms (*n* = 398, SMD = −0.54; Haller et al., 2021). DBT has also been recognized for its positive results. Warner and Murphy (2022) found that 78% of nine studies reported that DBT reduced alcohol and drug use in adults (*n* = 663). In addition, MBCT showed better short‐term results compared to CBT on quality of life (*n* = 161, SMD = 0.43; Haller et al., 2021). In younger populations, numerous scientific reviews have demonstrated the efficacy of mindfulness in general in a variety of clinical contexts. Firstly, Sun and colleagues (2021) reported positive effects of mindfulness on children's social‐emotional development (*n* = 3584). This literature review revealed that 13 of 16 studies (81%) found improvements in behavioral self‐regulation and executive function (Sun et al., 2021). Lee et al. (2022) also evaluated the effect of MBIs in reducing ADHD symptoms in children (*n* = 295, effect size = 0.77, *p* = 0.006; Lee et al., 2022). In addition, Kothgassner et al. (2021) found that DBT had weak to moderate effects for reducing self‐harm (effect size = −0.44, *p* = 0.021) and suicidal ideation (effect size = −0.31) in children (*n* = 1673). Collectively, these studies underline the efficacy of mindfulness therapies across a range of age groups and mental health contexts.

MBIs have shown the potential for effectively managing a wide range of mental health issues, including the management of tics, which are known to be intensified by stress, anxiety, and fatigue (Conelea & Woods, 2008). The nature of MBIs in fostering greater self‐awareness and emotional control (Kabat‐Zinn, 2003) aligns well with the requirements for managing CTD and Gilles de la TD. Tics, often exacerbated by psychological stressors, may be mitigated through the stress‐reducing techniques inherent in mindfulness practices. Moreover, the focused attention cultivated through MBIs mirrors the benefits seen in activities like playing an instrument or dancing, which require sustained attention and have been observed to attenuate tics, indicating a possible role for these interventions in tic regulation (Conelea & Woods, 2008).

The application of MBIs in the context of CTD and TD can be conceptualized through a logic model that charts the progression from structured program inputs—like mindfulness meditation, body awareness, and cognitive‐behavioral strategies. This can be through activities that include regular mindfulness sessions and cognitive reframing exercises. These activities lead to key outputs such as increased self‐awareness, improved emotional regulation, and enhanced attentional control. Such outputs then translate into intermediate outcomes, like heightened awareness and management of premonitory urges, reduced reactivity to stressors, and improved coping strategies for managing tics. Ultimately, these intermediate outcomes are expected to lead to long‐term outcomes encompassing a reduction in tic severity and frequency, an enhanced quality of life, and improved psychosocial functioning.

### 1.4 Why it is important to do this review

The need for this systematic review is highlighted by the existing gap in the evaluation of mindfulness‐based therapies for CTD and TD. While there are studies and pilot projects exploring the efficacy of mindfulness‐based techniques for these conditions, they do not constitute comprehensive systematic reviews. The existing literature on treatments for TD and CTD, including behavioral therapies like HRT, CBIT, and ERP, mainly focus on these specific interventions without a thorough exploration of mindfulness‐based approaches. Moreover, systematic reviews covering various treatments for tics typically synthesize evidence on medical, behavioral, and neurostimulation treatments, without a dedicated focus on mindfulness‐based therapies.

Given the potential of MBIs to address exacerbating factors of tics and their proven efficacy in managing stress, anxiety, and attentional issues (Haller et al., 2021; Sun et al., 2021), there is a compelling rationale for a systematic exploration and review of the effectiveness of MBIs in the treatment of CTD and TD. This exploration is not only crucial for filling the existing therapeutic gap but also for providing a comprehensive, holistic approach to managing these complex neurodevelopmental difficulties, offering the possibility of a more nuanced and strategic management of premonitory sensations and tics. Such a comprehensive approach underscores the value of mindfulness in potentially empowering individuals with CTD or TD to better manage their symptoms, paving the way for a holistic tic management strategy.

Current guidelines recommend combining pharmacotherapy with behavioral interventions as first‐line treatments to reduce the manifestation of tics (Andrén et al., 2022; Pringsheim et al., 2019). Individualized treatment options are recommended, and the decision should be a collaborative effort involving the patient, caregiver, and clinician (Pringsheim et al., 2019). However, not all behavioral interventions are effective for everyone experiencing tics (Scahill et al., 2013; Wilhelm et al., 2012). Given these challenges, MBIs present a promising addition to current guidelines. These interventions offer a cost‐effective and accessible option, potentially enhancing patient outcomes and satisfaction, especially for those adversely affected by current treatments (Scahill et al., 2013; Wilhelm et al., 2012). Such recommendations could involve healthcare policy changes, training for professionals in mindfulness interventions, and the development of patient education materials. By exploring these options, the review seeks to address treatment gaps for CTD and TD, offering timely insights to policymakers, clinicians, and patients, and advocating for a more holistic, patient‐centered approach to manage these difficulties.

## 2 OBJECTIVES

This systematic review aims to summarize the available scientific literature on the effect of mindfulness‐based approaches in children and adults with CTD or TD. More specifically, the research question is: What is the effectiveness of MBIs in reducing tic severity and improving overall functioning, quality of life, and co‐occurring symptoms in children and adults with CTD or TD?

## 3 METHODS

Follow Campbell Author guidelines (MECCIR conduct and reporting standards).

Cite the protocol at the review stage.

### 3.1 Criteria for considering studies for this review

#### 3.1.1 Types of studies and settings

We have selected randomized controlled trials (RCT), quasi‐experimental designs (QED), single‐group pre‐posttest (SGPP) designs, and single subject designs (SSD) for inclusion in this review. For the RCTs and QEDs, acceptable comparison groups encompass wait list control, no treatment, treatment‐as‐usual, and alternative treatments. Particularly for SSD studies, only those comparing alternating treatments or multiple baseline designs will be considered. RCTs are included for their strength in establishing causality with minimal bias. QEDs, while not as robust as RCTs, are often necessary in mindfulness research where randomization is not feasible as they provide valuable insights. SGPP designs are included due to ethical or practical challenges in using control groups in preliminary studies or specific contexts, allowing for initial evaluation of intervention effects. Finally, SSDs are important for understanding individual differences and tailoring interventions, offering detailed data on individual responsiveness. Our aim is to describe mindfulness approaches in various settings and to shed light on the methodologies and extents of intervention assessments. By including these diverse designs, we ensure that our review captures a wide array of studies, thus providing a broader understanding of how mindfulness interventions operate across different settings and populations. We acknowledge and will explore the limitations or biases associated with weaker designs, thereby providing a balanced and critical analysis of the existing literature.

#### 3.1.2 Types of participants

This review will include children (ages 5–17) and adults (ages 18 and above) who have been diagnosed with CTD or TD using recognized diagnostic criteria. We focus on individuals aged 5 and older as CTD and TD are typically not diagnosed before this age. As the effects of MBIs may differ based on whether the sample is comprised of participants that are high risk, studies involving participants with co‐morbid disorders will not be excluded. Furthermore, there are no restrictions based on the duration of diagnosis. As we are interested in informing intervention policy, studies that include participants in a variety of settings, ranging from clinical environments to community settings, will be included in this review. Studies whereby parents or teachers of children are the primary recipients of the intervention will be excluded.

#### 3.1.3 Types of interventions

Interventions of interest include those that use a mindfulness‐based approach. Mindfulness has its origins in ancient Eastern traditions and enables individuals to carefully observe their thoughts, emotions, and bodily sensations (Kabat‐Zinn, 1982). These practices not only improve self‐awareness, but also promote emotional regulation and adaptability (Kabat‐Zinn, 1982). Some specific interventions eligible for the study, include, but are not limited to, MBSR, DBT, ACT and MBCT. MBSR combines body awareness with yoga practices to reduce stress and improve overall well‐being (Kabat‐Zinn, 1990, 2003). DBT uses mindfulness to develop emotional regulation and interpersonal skills (Heard & Linehan, 1994), and ACT uses mindfulness to promote acceptance and behavior change (Hayes et al., 1999). Lastly, MBCT combines mindfulness practices with CBT (Morgan, 2003).

For the purpose of this systematic review, an MBI must include the following components to be considered an MBI:

1. The intervention must incorporate formal mindfulness practices, such as meditation, mindful breathing, body scan exercises, or mindful movement (e.g., yoga). These practices should aim to cultivate present‐moment awareness and non‐judgmental observation of thoughts, emotions, and bodily sensations.
2. The intervention should follow a structured program that includes regular sessions (e.g., weekly) over a specified period (e.g., 8 weeks). Each session should involve guided mindfulness practices and related activities.

In this systematic review, we will examine studies that have investigated MBIs, comparing them against various control conditions. The primary comparison condition will be “Treatment as Usual,” where participants receive standard care for their condition, excluding mindfulness‐based therapies. This comparison is crucial as it offers insight into the effectiveness of mindfulness interventions against the standard care practices. Another key comparison condition will involve studies that used active control groups. These groups participate in activities structurally similar to mindfulness interventions, such as relaxation techniques or health education classes, but do not include the mindfulness aspect. This comparison is vital to differentiate the specific effects of mindfulness from those of general therapeutic engagement or relaxation. Waitlist control groups will also be considered, where participants are initially not provided the mindfulness intervention but are later offered it post‐study. This condition helps in understanding the immediate effects of the intervention as compared to a delayed or no‐intervention scenario. Furthermore, studies that compare MBIs with specific non‐mindfulness therapies, like traditional CBT or psychoeducation, will also be included. This will allow us to assess how mindfulness‐based therapies perform in comparison to other established therapeutic approaches.

Exclusion criteria include studies that do not specifically focus on mindfulness‐based approaches, such as those involving only standard psychotherapy or pharmacotherapy without a mindfulness component. This exclusion is justified because the aim of the review is to assess the unique contributions of mindfulness practices in therapeutic interventions.

#### 3.1.4 Types of outcome measures

##### Primary outcomes

- Tic severity, measured at baseline and at least one time post‐intervention using standardized instruments.

##### Secondary outcomes

- Clinical improvement, which encompasses reductions in tic severity, improvements in quality of life, and reductions in the need for medication.
- The effectiveness of mindfulness interventions assessed using standardized tools in terms of clinical outcomes. This includes any additional outcomes reported by authors, provided they are assessed with standardized tools.
- Functioning, which includes the assessment of daily living skills, social functioning, academic or occupational performance, and overall quality of life. Specific scales and observational measures relevant to these domains will be identified and used.

- Severity of co‐occurring disorders, as the prevalence of comorbid conditions such as ADHD, OCD, anxiety, and depression in CTD and TD populations. The severity of these disorders will be measured using appropriate tools.

##### Additional considerations

- The review will also investigate potential negative outcomes or side effects associated with MBIs to provide a comprehensive safety profile.
- Changes in stress levels or other indirect effects of the intervention will be considered, as they play a significant role in the overall impact of the treatment for individuals with CTD and TD.

#### 3.1.5 Duration of follow‐up

This review will include studies with various follow‐up durations to capture both immediate and long‐term effects of mindfulness interventions, ranging from immediate post‐treatment to long‐term follow‐ups of a year or more. Studies lacking adequate follow‐up duration to assess sustained intervention effects will be excluded.

### 3.2 Search methods for identification of studies

#### 3.2.1 Electronic searches

Our search strategy is designed to comprehensively capture studies related to Tourette's Disorder and mindfulness interventions, conducted in both English and French. We will search electronic databases including Embase (1996–2024), PsychInfo (1806–2024), Global Health (1973–2024), PubMed (1975–2024), and Érudit (2000–2024). To complement our search, we will also use Ovid MEDLINE (1946–2024) for its advanced search capabilities, particularly with the use of MeSH terms, which can provide a more focused and precise retrieval of literature sources.

Our search terms, aligned with PICO criteria, will include specific keywords and their variations: “Tics,” “Tourette* Syndrome,” “Chronic Tic Disorder,” “mindfulness,” “Mindfulness‐Based Stress Reduction (MBSR),” “Dialectical Behavior Therapy (DBT),” “Acceptance and Commitment Therapy (ACT),” “Mindfulness‐Based Cognitive Therapy (MBCT),” as well as abbreviations such as “MBI” for Mindfulness‐Based Interventions, and “MCBT” for Mindfulness‐Based Cognitive Behavioral Therapy. We will enhance our search by including MeSH terms such as “Tic Disorders,” “Tourette Syndrome,” “Behavioral Symptoms,” “Mindfulness,” and “Cognitive Behavioral Therapy,” as well as additional specific subject headings such as “Self‐Compassion” under the broader term “Mindfulness.” An example of a detailed search for MEDLINE is: (“Tourette Syndrome”[MeSH] OR “Tic Disorders”[MeSH] OR tourette* OR tics OR “motor tics” OR “vocal tics”) AND (mindfulness OR “mindful meditation” OR “body scanning” OR “breath awareness” OR “mindfulness training” OR “Mindfulness‐Based Stress Reduction” OR “Mindfulness‐Based Cognitive Therapy” OR “Dialectical Behavior Therapy” OR “Acceptance and Commitment Therapy” OR “mindfulness meditation therapy” OR “mindfulness‐based stress therapy” OR “mindfulness‐based art therapy”) AND (“symptom reduction” OR “quality of life” OR “stress reduction” OR “coping strategies” OR “behavioral outcomes”) AND (intervention OR therapy OR treatment OR efficacy).

To ensure no potential research is missed, our strategy will also include searches of gray literature through ClinicalTrials.gov, WHO International Clinical Trials Registry Platform, ProQuest Dissertations & Theses, and OpenGrey. We will include conference proceedings from key psychology and neurology conferences, which will be identified based on their relevance to tics and mindfulness. Each gray literature source will be searched using tailored strategies that align with our main search terms and objectives.

#### 3.2.2 Searching other resources

When relevant, search trials registers and repositories of results, where relevant to the topic should be considered, as well as relevant gray literature sources such as reports/dissertations/theses databases and databases of conference abstracts, and reference lists of included studies and any relevant systematic reviews identified. Searching by contacting relevant individuals and organizations may also be considered.

### 3.3 Data collection and analysis

#### 3.3.1 Description of methods used in primary research

##### Selection of studies

Two independent reviewers will be involved in all stages of the search and selection process. All articles will be collected in the database and uploaded via the Zotero reference management software. The first step will be to remove all duplicate articles using Zotero's deduplication tool. Next, the titles and abstracts of all articles identified in the literature searches will be reviewed in Zotero for eligibility. Articles will be reviewed to determine whether the PICO eligibility matches the inclusion criteria. Potential studies will either be identified and noted as potentially eligible, retained in Zotero, or deleted if deemed ineligible. After retrieving the full texts of appropriate studies marked as potentially eligible, the full texts will be evaluated. Each article will be reviewed, and relevant data will be extracted.

##### Data extraction and management

Data from the included studies will be obtained using data extraction tables. First, descriptive information such as study reference, country, study type, population, number of participants, sex ratio, duration of intervention and follow‐up will be extracted. Outcome measures will then be extracted in separate tables.

##### Assessment of risk of bias in included studies

To assess study quality and risk of bias, two independent reviewers will employ the Cochrane Risk of Bias Tool (RoB‐II) for randomized trials and the Risk Of Bias In Non‐randomized Studies of Interventions (ROBINS‐I) for non‐randomized studies. Each study will be categorized into low, high, or unclear risk of bias based on selection, performance, detection, attrition, and reporting biases. In cases where studies differ from traditional randomized or non‐RCT designs, particularly with SSDs and SGPP designs, we will adopt qualitative approaches and narrative synthesis. A qualitative examination of each study will be done, focusing on its context, methodology, and the implications these aspects have on bias risk. To facilitate this assessment, we will develop a coded table that integrates adapted headings from established risk of bias tools.

##### Measures of treatment effect

In this systematic review, we will calculate and interpret effect sizes from individual studies to assess the impact of mindfulness interventions on tic severity and related outcomes. The methodologies for calculating effect sizes vary depending on the study design.

For RCTs and QEDs with control groups, we will calculate standardized mean differences (Cohen's *d* or Hedges' *g*) for continuous outcomes, and risk ratios or odds ratios for categorical outcomes between groups. For SGPPs, we will calculate Cohen's d to measure the effect of the intervention within the same group. For SSDs, we will use single‐case methods, such as the improvement rate difference, to observe individual responses to the intervention. If data for calculating effect sizes is missing, we will contact study authors for additional information. If the data remains unavailable, the studies will be included in the narrative synthesis with a discussion on the impact of the missing data.

##### Unit of analysis issues

In addressing unit‐of‐analysis issues, such as clustering, crossover designs, and multiple outcome measurements, we will adhere to qualitative guidelines provided in the Cochrane Handbook, particularly chapters 6 and 23. Our focus will be on narratively synthesizing how these issues impact the findings, rather than on quantitative adjustments.

##### Criteria for determination of independent findings

In our systematic review, we aim to maintain methodological integrity when handling dependencies such as multiple reports from the same study and similar outcomes reported within studies. We will select the most comprehensive report from multiple accounts of the same study to prevent data duplication. For outcomes reported in various formats within the same study, each will be documented and assessed separately in our narrative synthesis. This approach ensures that no quantitative pooling of similar outcomes is implied, respecting the distinctiveness of each data presentation. All decisions related to data handling will be systematically documented and justified. Regarding missing data, efforts will be made to contact study authors for necessary clarifications; however, studies with critical unresolved data will be discussed for their potential impact but will not be included in detailed analysis. This process aligns with our commitment to providing a thorough, narrative‐driven exploration of the effectiveness of mindfulness interventions in managing tic disorders.

##### Assessment of heterogeneity

Variations in study designs, participant characteristics, and interventions will be discussed qualitatively. This narrative synthesis will highlight the diversity and comparability of the studies.

##### Assessment of reporting biases

We will qualitatively assess the potential for reporting biases, such as publication bias, by examining the characteristics of the included studies and their reporting trends.

##### Data synthesis

Narrative synthesis will be used to summarize and explain the results of the review, with a comparison of results between studies. This process involves the use of words and text to summarize and explain the results of several studies. The main feature of this synthesis is the use of a written approach to describe the overall effects of the studies considered (Popay et al., 2006). The first step in the synthesis will be to familiarize ourselves with the results of the studies by systematically evaluating the findings and highlighting important features. Next, study data will be collected and grouped for comparison, so that studies are grouped according to the outcome measure used and whether they involve children or adults. The focus is on assessing clinically relevant outcomes of MBIs for CTD or TD.

In preparing this protocol, we have considered the SWiM (Synthesis Without Meta‐analysis) guidelines to guide our narrative synthesis approach. Given the heterogeneity expected in study designs, interventions, and outcomes, as well as the small number of studies, a narrative synthesis allows for a more flexible and nuanced exploration of the effects of MBIs for CTDs and TDs. This approach is chosen to accommodate the anticipated variability in methodologies and clinical settings of the included studies, ensuring a comprehensive understanding of the intervention effects across different populations and contexts.

We will employ a stratified synthesis approach to accommodate the inclusion of various study designs such as RCTs, QEDs, SGD, and SSDs. Recognizing the methodological differences between these study types, we will synthesize the results of RCTs separately from QEDs, SGDs, and SSDs to maintain the integrity of evidence levels. Each study type will be assessed for its risk of bias and its contribution to the overall evidence base.

Given the range of study designs included, our narrative synthesis will arrange findings to respect the unique contributions and methodological considerations of each study type. We will synthesize the results of different study designs separately to provide a clear and nuanced understanding of the evidence base.

##### Subgroup analysis and investigation of heterogeneity

Differences observed across various subgroups within the studies will be described narratively. Subgroup analyses will be conducted based on predetermined criteria, such as age, type of intervention, and coexisting conditions. We will explore and qualitatively report on the heterogeneity observed within and between these subgroups to understand their influence on the intervention effects.

##### Sensitivity analysis

Sensitivity analyses will be narratively described, outlining how variations in methodological choices, such as risk of bias or sample size, could potentially affect the robustness and validity of our review findings.

##### Treatment of qualitative research

Qualitative research, which typically uses different methodologies and may not provide quantifiable outcomes, falls outside the scope of this specific review.

##### Summary of findings and assessment of the certainty of the evidence

The final section will present a summary of the key findings and a qualitative assessment of the overall certainty and quality of the evidence, based on the comprehensiveness and consistency of the reported results.

## CONTRIBUTIONS OF AUTHORS

### Content

Julie B. Leclerc, MPs, PhD, is ideally suited for the role of content expert. Julie specializes in the study of childhood psychopathology and Tourette Disorder. She works to implement evidence‐based interventions. She is director of mental health research at CIUSSS du Nord‐de‐l'Île‐de‐Montréal and a full professor in the psychology department at Université du Québec à Montréal. She is also a member of the Réseau intersectoriel de recherche en santé de l'Université du Québec and an associate researcher at the Institut universitaire en santé mentale de Montréal. With her specialization in childhood psychopathology and Tourette's Disorder, combined with her experience in implementing evidence‐based interventions, she brings a deep understanding of the subject matter. Her positions as a director of mental health research and a full professor in psychology, as well as her involvement with various research networks, further solidify her expertise in this area.

### Systematic review methods

Ilana Singer, MSc, is well‐positioned to contribute to systematic review methods. Her education and research experience at King's College London and Université du Québec à Montréal, particularly her involvement in diverse research projects and coordination roles, demonstrate a strong grasp of systematic methodologies. Ilana's role as a research coordinator and her experience in conducting systematic reviews and academic research align well with the demands of a systematic review.

### Statistical analysis

Méliza Gagnon, BSc, a fourth‐year PhD student in clinical psychology at the Université du Québec à Montréal, possesses crucial statistical analysis skills. Her experience tutoring in quantitative data analysis and her role as a research assistant dealing with complex data sets make her well‐prepared for the statistical requirements of the systematic review.

Ilana Singer, MSc, as a second‐year PhD student in clinical psychology at the Université du Québec à Montréal, significantly enhances the team's capacity for statistical analysis. Her health psychology background from King's College London and proficiency with statistical software like SPSS and STATA are complemented by her experience conducting thematic analysis during her master's. This combination of skills and experiences makes her particularly well‐suited for managing the advanced data analysis and synthesis needed for the review.

### Information retrieval

Ilana Singer, MSc, and Méliza Gagnon, BSc, are both suitable for the role of information retrieval, given their extensive academic background and research experience. Their proficiency with data management tools and familiarity with academic reference organization tools like Zotero will be beneficial in efficiently sourcing and organizing relevant literature and data for the review.

Each team member's specific educational background and professional experience effectively cover the essential areas needed for a comprehensive and methodologically sound systematic review. This team composition ensures a balanced approach, combining content expertise, methodological rigor, statistical proficiency, and efficient information retrieval capabilities.

## DECLARATIONS OF INTEREST

Author Julie B. Leclerc, MPs, PhD, has been involved in the creation of the *Cognitive Psychophysiological treatment* (CoPs) for Tourette's Disorder (Leclerc et al., 2016).

### Preliminary timeframe

The approximate date for submission of the systematic review will be 12 months after protocol approval.

### Plans for updating this review

The review will be updated biennially by the lead author, Ilana Singer, in collaboration with the research team. The process will involve a comprehensive re‐evaluation of the literature and incorporation of recent findings to ensure the review remains current and reflective of the latest research and practices in the field.

### SOURCES OF SUPPORT

### Internal sources

- No sources of support provided.

### External sources

- No sources of support provided.

## REGISTRATION AND PROTOCOL

Title Registration: Singer, I., Gagnon, M., & Leclerc, J. (2023). Mindfulness‐based interventions for improving tic‐related symptoms in children and adults with chronic tic disorder and Tourette's disorder: A systematic review. Cochrane Database of Systematic Reviews. Art. No.: CA000421.

### What's new

**Table:** 

Date	Event	Description
18 December 2023	Amended	

## Supporting information

Supporting information.

Supporting information.

Supporting information.

Supporting information.

Supporting information.

Supporting information.

Supporting information.

Supporting information.

Supporting information.

Supporting information.

## References

[cl21427-bib-0001] Allen, J. G. , Romate, J. , & Rajkumar, E. (2021). Mindfulness‐based positive psychology interventions: A systematic review. BMC Psychology, 9(1), 116. 10.1186/s40359-021-00618-2 34362457 PMC8344333

[cl21427-bib-0002] American Psychiatric Association . (2022). Diagnostic and Statistical Manual of Mental Disorders (DSM‐5‐TR). American Psychiatric Association Publishing. 10.1176/appi.books.9780890425787

[cl21427-bib-0003] Andrén, P. , Jakubovski, E. , Murphy, T. L. , Woitecki, K. , Tarnok, Z. , Zimmerman‐Brenner, S. , Van De Griendt, J. , Debes, N. M. , Viefhaus, P. , Robinson, S. , Roessner, V. , Ganos, C. , Szejko, N. , Müller‐Vahl, K. R. , Cath, D. , Hartmann, A. , & Verdellen, C. (2022). European clinical guidelines for Tourette syndrome and other tic disorders—Version 2.0. Part II: Psychological interventions. European Child & Adolescent Psychiatry, 31(3), 403–423. 10.1007/s00787-021-01845-z 34313861 PMC8314030

[cl21427-bib-0004] Conelea, C. A. , & Woods, D. W. (2008). The influence of contextual factors on tic expression in Tourette's syndrome: A review. Journal of Psychosomatic Research, 65(5), 487–496. 10.1016/j.jpsychores.2008.04.010 18940379

[cl21427-bib-0005] Cox, J. H. , Nahar, A. , Termine, C. , Agosti, M. , Balottin, U. , Seri, S. , & Cavanna, A. E. (2019). Social stigma and self‐perception in adolescents with Tourette syndrome. Adolescent Health, Medicine and Therapeutics, 10, 75–82. 10.2147/AHMT.S175765 31354374 PMC6573773

[cl21427-bib-0006] Dimidjian, S. , Arch, J. J. , Schneider, R. L. , Desormeau, P. , Felder, J. N. , & Segal, Z. V. (2016). Considering meta‐analysis, meaning, and metaphor: A systematic review and critical examination of “Third Wave” cognitive and behavioral therapies. Behavior Therapy, 47(6), 886–905. 10.1016/j.beth.2016.07.002 27993339

[cl21427-bib-0007] Eapen, V. , Cavanna, A. E. , & Robertson, M. M. (2016). Comorbidities, social impact, and quality of life in Tourette syndrome. Frontiers in Psychiatry, 7, 97. 10.3389/fpsyt.2016.00097 27375503 PMC4893483

[cl21427-bib-0008] Gill, C. E. , & Kompoliti, K. (2020). Clinical features of Tourette syndrome. Journal of Child Neurology, 35(2), 166–174. 10.1177/0883073819877335 31608744

[cl21427-bib-0009] Haller, H. , Breilmann, P. , Schröter, M. , Dobos, G. , & Cramer, H. (2021). A systematic review and meta‐analysis of acceptance‐ and mindfulness‐based interventions for DSM‐5 anxiety disorders. Scientific Reports, 11(1), 20385. 10.1038/s41598-021-99882-w 34650179 PMC8516851

[cl21427-bib-0010] Hayes, S. C. , Strosahl, K. , & Wilson, K. G. (1999). Acceptance and Commitment Therapy: An experiential approach to behavior change. Guilford Press.

[cl21427-bib-0011] Heard, H. L. , & Linehan, M. M. (1994). Dialectical behavior therapy: An integrative approach to the treatment of borderline personality disorder. Journal of Psychotherapy Integration, 4(1), 55–82. 10.1037/h0101147

[cl21427-bib-0012] Kabat‐Zinn, J. (1982). An outpatient program in behavioral medicine for chronic pain patients based on the practice of mindfulness meditation: Theoretical considerations and preliminary results. General Hospital Psychiatry, 4(1), 33–47. 10.1016/0163-8343(82)90026-3 7042457

[cl21427-bib-0013] Kabat‐Zinn, J. (1990). Full catastrophe living. Using the wisdom of your body and mind to face stress, pain and illness. Bantam Doubleday Dell Publishing.

[cl21427-bib-0014] Kabat‐Zinn, J. (2003). Mindfulness‐based interventions in context: Past, present, and future. Clinical Psychology: Science and Practice, 10(2), 144–156. 10.1093/clipsy.bpg016

[cl21427-bib-0015] Knight, T. , Steeves, T. , Day, L. , Lowerison, M. , Jette, N. , & Pringsheim, T. (2012). Prevalence of tic disorders: A systematic review and meta‐analysis. Pediatric Neurology, 47(2), 77–90. 10.1016/j.pediatrneurol.2012.05.002 22759682

[cl21427-bib-0016] Kothgassner, O. D. , Goreis, A. , Robinson, K. , Huscsava, M. M. , Schmahl, C. , & Plener, P. L. (2021). Efficacy of dialectical behavior therapy for adolescent self‐harm and suicidal ideation: A systematic review and meta‐analysis. Psychological Medicine, 51(7), 1057–1067. 10.1017/S0033291721001355 33875025 PMC8188531

[cl21427-bib-0016a] Leclerc, J. B. , Valois, P. , J‐Nolin, G. , Bombardier, M. , Ouellette, S. , & O'Connor, K. P. (2016). A therapy for tics in children managing underlying processes: A pilot study. Journal of Developmental and Physical Disabilities, 28(4), 581–593. 10.1007/s10882-016-9496-y

[cl21427-bib-0017] Lee, Y.‐C. , Chen, C.‐R. , & Lin, K.‐C. (2022). Effects of mindfulness‐based interventions in children and adolescents with ADHD: A systematic review and meta‐analysis of randomized controlled trials. International Journal of Environmental Research and Public Health, 19(22), 15198. 10.3390/ijerph192215198 36429915 PMC9690476

[cl21427-bib-0018] Morgan, D. (2003). Mindfulness‐based cognitive therapy for depression: A new approach to preventing relapse. Psychotherapy Research, 13(1), 123–125. 10.1080/713869628 22475168

[cl21427-bib-0019] Nolin, G. J. , & Leclerc, J. B. (2021). Articles libres. Revue Québécoise de Psychologie, 42(1), 171–194. 10.7202/1078922ar

[cl21427-bib-0019a] Popay, J. , Roberts, H. , Sowden, A. , Petticrew, M. , Arai, L. , Rodgers, M. , Britten, N. , Roen, K. , & Duffy, S. (2006). Guidance on the conduct of narrative synthesis in systematic reviews: A product from the ESRC methods programme. Lancaster University. 10.13140/2.1.1018.4643

[cl21427-bib-0020] Pringsheim, T. , Okun, M. S. , Müller‐Vahl, K. , Martino, D. , Jankovic, J. , Cavanna, A. E. , Woods, D. W. , Robinson, M. , Jarvie, E. , Roessner, V. , Oskoui, M. , Holler‐Managan, Y. , & Piacentini, J. (2019). Practice guideline recommendations summary: Treatment of tics in people with Tourette syndrome and chronic tic disorders. Neurology, 92(19), 896–906. 10.1212/WNL.0000000000007466 31061208 PMC6537133

[cl21427-bib-0021] Scahill, L. , Woods, D. W. , Himle, M. B. , Peterson, A. L. , Wilhelm, S. , Piacentini, J. C. , McNaught, K. , Walkup, J. T. , & Mink, J. W. (2013). Current controversies on the role of behavior therapy in Tourette syndrome. Movement Disorders, 28(9), 1179–1183. 10.1002/mds.25488 23681719 PMC4283569

[cl21427-bib-0021a] Segal, Z. , Williams, M. , & Teasdale, J. (2002). Mindfulness‐based cognitive therapy for depression: A new approach to preventing relapse: Book review. Cognitive Behaviour Therapy, 31(4), 193–194.

[cl21427-bib-0022] Solís García, G. , Jové Blanco, A. , Chacón Pascual, A. , Vázquez López, M. , Castro De Castro, P. , Carballo Belloso, J. J. , Pina Camacho, L. , & Miranda Herrero, M. C. (2021). Calidad de vida y comorbilidades psiquiátricas en pacientes pediátricos con síndrome de Gilles de la Tourette. Revista de Neurología, 73(10), 339. 10.33588/rn.7310.2021046 34755886

[cl21427-bib-0023] Sun, Y. , Lamoreau, R. , O'Connell, S. , Horlick, R. , & Bazzano, A. N. (2021). Yoga and mindfulness interventions for preschool‐aged children in educational settings: A systematic review. International Journal of Environmental Research and Public Health, 18(11), 6091. 10.3390/ijerph18116091 34198737 PMC8201280

[cl21427-bib-0024] Warner, N. , & Murphy, M. (2022). Dialectical behaviour therapy skills training for individuals with substance use disorder: A systematic review. Drug and Alcohol Review, 41(2), 501–516. 10.1111/dar.13362 34337811

[cl21427-bib-0025] Wilhelm, S. , Peterson, A. L. , Piacentini, J. , Woods, D. W. , Deckersbach, T. , Sukhodolsky, D. G. , Chang, S. , Liu, H. , Dziura, J. , Walkup, J. T. , & Scahill, L. (2012). Randomized trial of behavior therapy for adults with Tourette syndrome. Archives of General Psychiatry, 69(8), 795. 10.1001/archgenpsychiatry.2011.1528 22868933 PMC3772729

[cl21427-bib-0026] Yang, J. , Hirsch, L. , Martino, D. , Jette, N. , Roberts, J. , & Pringsheim, T. (2016). The prevalence of diagnosed Tourette syndrome in Canada: A national population‐based study. Movement Disorders, 31(11), 1658–1663. 10.1002/mds.26766 27548401

